# Genetic and antigenic characterization of serotype O FMD viruses from East Africa for the selection of suitable vaccine strain

**DOI:** 10.1016/j.vaccine.2017.10.040

**Published:** 2017-12-14

**Authors:** Katie Lloyd-Jones, Mana Mahapatra, Sasmita Upadhyaya, David J. Paton, Aravindh Babu, Geoff Hutchings, Satya Parida

**Affiliations:** a The Pirbright Institute, Ash Road, Pirbright, Surrey GU24 ONF, UK; b National Institute for Animal Biotechnology, Hyderabad 500049, India

**Keywords:** East Africa, FMD, Serotype O, Vaccine strain selection

## Abstract

Foot-and-mouth disease (FMD) is endemic in Eastern Africa with circulation of multiple serotypes of the virus in the region. Most of the outbreaks are caused by serotype O followed by serotype A. The lack of concerted FMD control programmes in Africa has provided little incentive for vaccine producers to select vaccines that are tailored to circulating regional isolates creating further negative feedback to deter the introduction of vaccine-based control schemes. In this study a total of 80 serotype O FMD viruses (FMDV) isolated from 1993 to 2012 from East and North Africa were characterized by virus neutralisation tests using bovine antisera to three existing (O/KEN/77/78, O/Manisa and O/PanAsia-2) and three putative (O/EA/2002, O/EA/2009 and O/EA/2010) vaccine strains and by capsid sequencing. Genetically, these viruses were grouped as either of East African origin with subdivision into four topotypes (EA-1, 2, 3 and 4) or of Middle-East South Asian (ME-SA) topotype. The ME-SA topotype viruses were mainly detected in Egypt and Libya reflecting the trade links with the Middle East countries. There was good serological cross-reactivity between the vaccine strains and most of the field isolates analysed, indicating that vaccine selection should not be a major constraint for control of serotype O FMD by vaccination, and that both local and internationally available commercial vaccines could be used. The O/KEN/77/78 vaccine, commonly used in the region, exhibited comparatively lower percent in vitro match against the predominant topotypes (EA-2 and EA-3) circulating in the region whereas O/PanAsia-2 and O/Manisa vaccines revealed broader protection against East African serotype O viruses, even though they genetically belong to the ME-SA topotype.

## 1 Introduction

Foot-and-mouth disease (FMD) remains a globally important livestock animal disease affecting cloven-hoofed domestic and wild animals. It is endemic in Africa and Asia, and seriously affects livestock productivity through decrease in milk yield, loss of draught power and weight loss [1]. In addition, strict international legislation preventing export of animals or animal products from FMD endemic countries causes severe economic hardship to farmers. The causative agent, foot-and-mouth disease virus (FMDV) exists as seven immunologically distinct serotypes, O, A, C, Asia 1, SAT (Southern African Territory) 1, 2 and 3, each with a wide spectrum of antigenically distinct subtypes. Serotype C virus has not been detected since 2004 [2], [3] and is probably extinct. FMDV is a single stranded positive sense RNA virus of the genus *Aphthovirus*, family *Picornaviridae*. The RNA is enclosed in a capsid that is made up of 60 copies of 4 different structural proteins (VP1-4); VP1-3 are exposed on the surface of the capsid whilst the VP4 is internal. Five neutralising antigenic sites have been identified on the surface of the capsid by sequencing monoclonal antibody (mAb) neutralisation resistant (mar) mutants in case of serotype O viruses [4].

In East Africa, attempts to control FMD have been mainly by vaccination to control outbreaks and sometimes for routine prophylaxis; efforts that are hampered by the occurrence of several serotypes and/or strains of FMDV. Although efforts to control FMD have been made for nearly 40 years there appears to be very limited success partly due to lack of tailored made vaccines against circulating viruses, and/or availability of vaccines of good quality and potency. Most FMD outbreaks in East Africa have been caused by serotype O, followed by serotype A and SAT2 [5]. Control of the disease mainly depends on availability of matching vaccines that can be selected based on epidemiological information and serological cross-reactivity of bovine post-vaccinal serum (BVS) with circulating viruses. In addition, availability of sufficient doses of vaccines of good quality and potency is also equally important. Mono-, bi- and quadri-valent vaccines are currently in use in East African countries for FMD control [6], [7]. These vaccines are mainly produced in vaccine production plants located in Ethiopia and Kenya using relatively historic viruses and rarely carrying out vaccine matching tests to select the best vaccine for use in the region [8]. Hence, the existing vaccines may not provide optimal protection against recently circulating FMD viruses. This study was, therefore, designed to characterise recently circulating serotype O FMD viruses in the region both antigenically and genetically and recommend matching vaccine strains (v/s) for use in FMD control programmes in East African countries.

## 2 Materials and methods

### 2.1 Cells and viruses

Eighty serotype O viruses from Africa submitted to the World Reference Laboratory for FMD (WRLFMD) at Pirbright were used in this study (Supplementary Table 1). One is the v/s, O/KEN/77/78 that was originally isolated from Kenya in 1978. The other 79 viruses were isolated over a 20-year period between 1993 and 2012. The viruses were from seven East African countries, Ethiopia (n = 29), Eritrea (n = 4), Sudan (n = 11), Kenya (n = 6), Somalia (n = 3), Tanzania (n = 3), Uganda (n = 3) and from five neighbouring/trade related countries: Democratic Republic of Congo (COD, n = 2), Egypt (n = 7), Libya (n = 9), Nigeria (n = 1) and Zambia (n = 2). In addition, two widely used serotype O vaccine viruses, O/Manisa and O/PanAsia-2 originally isolated from Turkey in 1969 and 2009, respectively, were also used in this study making a total of 82 viruses. These samples were derived from cattle epithelial tissues except five viruses from Ethiopia, one from Libya and one from Uganda whose host species is not known (Supplementary Table 1). In addition, one virus each from Eritrea and Ethiopia were isolated from pigs; and one virus from Egypt and two viruses from Libya were isolated from sheep. All samples were initially grown in primary bovine thyroid cells (BTY) with subsequent passage in either BHK-21 or IB-RS2 cells. Stocks of virus were prepared by infecting IB-RS2 cell monolayers and were stored as clarified tissue culture harvest material at −70 °C until required.

### 2.2 Polyclonal sera

Six bovine anti-FMDV polyclonal sera were used in the study. Three of these, had been raised against existing v/s, namely O/KEN/77/78, O/Manisa and O/PanAsia-2 as previously described [9] whereas the other three are against putative v/s. The antisera against the putative strains (two, O/EA/2002 and O/EA/2009 from EA-2 topotype and one, O/EA/2010 from EA-3 topotype) were raised in cattle at Pirbright as described previously [10] by administering inactivated, purified 146S FMD virus particles in ISA-206 adjuvant. The animals were boosted on 21-day post-vaccination and bled one week later for preparation of sera. For each virus, a pool of sera from five animals was used in the serological tests. The homologous neutralising antibody titres of each pooled serum were in the range of log_10_ 2.1 to 2.5 (data not shown).

### 2.3 Two-dimensional micro-neutralisation test (2D-VNT)

The 2D-VNT test was carried out using the pooled post-vaccination bovine sera according to Rweyemamu et al. [11]. Antibody titres were calculated from regression data as the log_10_ reciprocal antibody dilution required for 50% neutralisation of 100 tissue culture infective units of virus (log_10_SN_50_/100 TCID_50_). The neutralising antigenic relationship of viruses is given by the ratio: ‘r_1_’ = neutralising antibody titre against the heterologous virus/neutralising antibody titre against the homologous virus. The significance of differences between ‘r_1_-values’ obtained by the polyclonal antiserum was evaluated according to standard criteria [12]. All the tests were repeated at least twice and the average of the two tests was used for further analysis.

### 2.4 Nucleotide (nt) sequencing and analysis of the sequence data

The sequences of the entire capsid coding region (P1) of the viruses were generated. RNA extraction from the cell culture grown viruses, reverse transcription (RT), polymerase chain reaction (PCR) to amplify the P1 region, sequencing, sequence analysis and assembling, and alignment were performed as described previously [13]. Nt sequences of the viruses were aligned using the CLUSTAL X multiple sequence alignment program [14] and the predicted amino acid (aa) sequences were translated using BioEdit 7.0.1 [15]. The alignments were used to construct distance matrices using the Kimura 2-parameter nucleotide substitution model [16] as implemented in the program MEGA 6.0 [17].

### 2.5 Genetic characterisation

The aligned complete P1 nt sequences were used to determine the most suitable nt substitution model using jModelTest [18] and MEGA [17] resulting in selection of the General time reversal (GTR) model with a combination of gamma distribution and proportion of invariant sites (GTR + G+I). Then, Bayesian analysis was performed using the BEAST software package v1.8.4 [19]. In BEAUti v1.8.4, the ages of the viruses were defined by the date of sample collection and the analysis used the GTR + G+I model to describe the rate heterogeneity among sites. Variations in substitution rate among branches were evaluated by comparing four different clocks in BEAST. The maximum clade credibility (MCC) phylogenetic tree was inferred using the Bayesian Markov Chain Monte Carlo (MCMC) method. Then, a Bayes factor analysis in TRACER version 1.6 [20] was used to determine the best-fit model that resulted in the selection of an uncorrelated exponential relaxed molecular clock. The tree was obtained using the Tree Annotator program in BEAST and the evolutionary trees were viewed in FigTree program 1.4.2.

### 2.6 Statistical analysis

The proportion of synonymous substitutions per potential synonymous site and the proportion of non-synonymous substitutions per potential non-synonymous site were calculated by the method of Nei and Gojobori [21] using the SNAP program (www.hiv.lanl.gov). The aa variability of the capsid region of the EA-type O viruses was determined essentially as described by Valdar [22]. The statistical analysis was carried out using Minitab release 12.21 software.

## 3 Results and discussion

Of the five main serotypes (O, A, SAT 1-3) circulating in Eastern Africa, most outbreaks are caused by serotype O followed by serotypes A and SAT-2 [5]. The main serotype O topotypes found in East Africa are EA-1, EA-2, EA-3 and EA-4 (Table 1A). In addition, another topotype from the Middle East, ME-SA (PanAsia-2) has been circulating in Egypt and Libya since 2007 and 2010, respectively, probably because of trade links with countries from the Middle East. Another strain of the ME-SA topotype, O-Ind-2001d, originating from the Indian sub-continent has also been detected in Libya since 2013. These O-Ind-2001d viruses have also spread to Tunisia, Algeria and Morocco (Table 1A, Table 1B). Historically, the EA-1 topotype has been prevalent in East Africa and accordingly three Kenyan v/s (KEN/120/64, KEN/77/78 and KEN/83/79) were developed of which KEN/77/78 has been regularly used in vaccination programmes for more than three decades in the region [6], [7]. However, recent epidemiological data reveals fewer EA-1 outbreaks and this topotype is now mostly restricted to Kenya only (Table 1A). A previous report also indicated that viruses of this topotype were mainly circulating in Kenya and Uganda during 1964–1996 [6]. Therefore, it is possible that this topotype may disappear, like serotype C, which was last detected in 2004. Similarly, EA-4 viruses were first detected in Ethiopia in 2005 and have been mostly restricted to Ethiopia with occasional involvement in outbreaks in Kenya in 2010. Currently, EA-2 and EA-3 are the predominant topotypes causing most of the serotype O outbreaks in the region (Table 1A).

**Table 1A:** Different topotypes of serotype O FMD viruses currently circulating in the East African countries employed in this study. In addition, Ind-2001d viruses of ME-SA topotype are also circulating in North African countries, Algeria (2014), Morocco (2015) and Tunisia (2014). PA-2: PanAsia-2; -: none reported.

Country/Topotypes	EA-1	EA-2	EA-3	EA-4	ME-SA
DRC	–	2006, 2010	–	–	–
Egypt	–	–	2012–16	–	2006–09, 2011 (Sharqia-72 and PA-2)
Eritrea	–	–	2011	–	–
Ethiopia	–	–	2004–15	2005, 2013, 2016	–
Kenya	2008–10	2004–05, 2007–11	–	2010	–
Libya	–	–	2012	–	2010–12 (PA-2); 2013 (Ind- 2001d)
Nigeria	–	–	2007, 2009, 2011	–	–
Somalia	–	–	2007	–	–
Sudan	–	–	2005, 2008–13	–	–
Tanzania	–	2008–09, 2012–14	–	–	–
Uganda	–	2007	–	–	–
Zambia	–	2010	–	–	–

### 3.1 Serological characterisation of the East African serotype O viruses

**Fig. 1:**
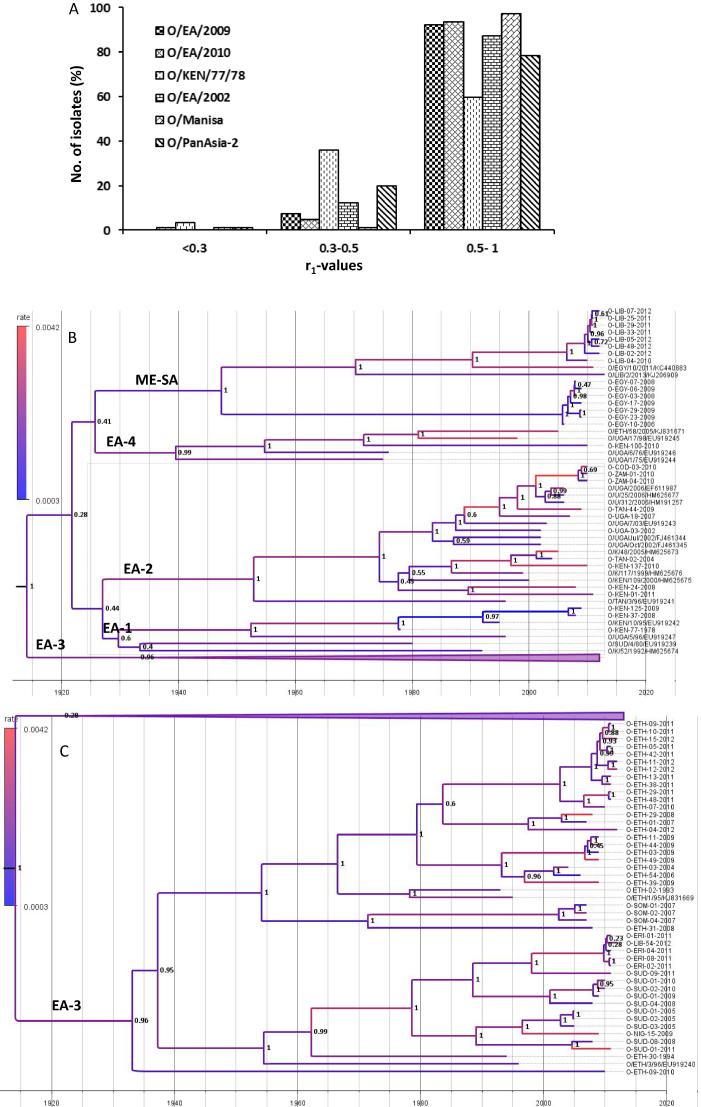
A: Antigenic relationship (r_1_) values of serotype O East African isolates against six post-vaccinal bovine antisera. The cut-off value is 0.3, above which the vaccine is considered to be a good match. (B and C): Bayesian phylogenetic tree of the East African serotype O viruses. The capsid sequences retrieved from Genbank are indicated by their respective accession numbers.

To our knowledge there are not many reports on the vaccine matching results involving serotype O viruses from Eastern Africa. In this study, the cross-reactivity of the East African serotype O viruses (n = 80) was measured by 2D-VNT using post-vaccination bovine sera against 6 vaccines. Out of these, O/KEN/77/78 is the only established vaccine originating from the region. The v/s originating from the Middle East, O/Manisa and O/PanAsia-2 were also included to test their suitability to be used as a vaccine in the region. In addition, antisera were generated against three putative strains representing the predominant topotypes (two from EA-2 and one from EA-3) circulating in the region (Supplementary Table 1). All the six antisera exhibited strong cross-reactivity with the viruses (>95%) indicating that these vaccines are a good match for the currently circulating field isolates (Fig. 1A). Considering serotype O is the most prevalent serotype in the region this is a significant finding as this provides reassurance that at least for serotype O, vaccine strain selection should not prove a significant constraint to FMD control in East Africa. Of the three established v/s O/Manisa and O/PanAsia-2 were more cross-reactive than the O/KEN/77/78 v/s (Fig. 1A). A relatively higher number of isolates (∼36%) exhibited r_1_-values in the range of 0.3–0.5 against O/KEN/77/78 (Fig. 1A) indicating regular vaccine matching work involving the circulating viruses needs to be carried out for continual use of this vaccine strain in the FMD control programmes in future. The percent in vitro protection (percentage of viruses exhibiting an r_1_-value more than 0.3) exhibited by each v/s with the different topotypes circulating in the region are shown in Table 1B. Interestingly, O/KEN/77/78 v/s also showed comparatively lower percent in vitro match against the predominant topotypes (EA-2 and EA-3) circulating in the region (Table 1B). Serotype O Ethiopian candidate v/s belonging to EA-3 and EA-4 topotype have been reported to be a good match (in vitro) with the serotype O Ethiopian isolates [23]. Therefore, it appears that the antigenicity of the type O viruses circulating in the region has not changed greatly, and the vaccines of Middle East origin can also be used in East Africa to control the disease. Indeed, Ethiopia has imported O/Manisa vaccine for use in vaccination programmes in the country. The Indian type O vaccine (O/IND/R2/75) has also been reported to cross-react with viruses from East Africa, mainly Eritrea, Ethiopia, Kenya and Sudan [24]. However in recent days it has increasingly become obvious that r_1_-values (in vitro) alone cannot reliably predict protection as has been demonstrated in several heterologous challenge experiments [25], [26]. To the best of our knowledge there is no published report on the heterologous challenge studies involving East-African type O viruses.

**Table 1B:** Percent (%) in vitro protection exhibited by different type O vaccines against the viruses of different topotypes circulating in East-Africa. The lineages are shown in parenthesis. SH72: Sharqia-72, PA: PanAsia.

Vaccine/topotype	O/EA/2009	O/EA/2010	O/KEN/77/78	O/Manisa	O/PanAsia-2
EA-1	100	66.6	100	100	100
EA-2	100	100	92.3	100	100
EA-3	100	100	97.9	100	100
EA-4	100	100	100	100	100
ME-SA (SH72)	100	100	100	100	100
ME-SA (PA)	100	100	100	100	100
Total	100	98.7	97.5	100	100

Because of the globalisation of trade and migration of people there is always a possibility of introduction of new lineages into new territory. O-Ind-2001d viruses have been circulating in North Africa since 2013 and have now spread to other North African countries (Table 1A), and could be a threat to East Africa. There is no published report on matching vaccine for O-Ind-2001d viruses; however it is envisaged that the serotype O vaccines would be able to provide protection against viruses of this lineage as it is well known that serotype O vaccines are broadly cross-reactive. Indeed our previous study revealed Indian v/s, O/IND/R2/75 to be a good in vitro match with viruses of this lineage isolated from India [24]. In addition, high antigen payload vaccines have been shown to compensate poor vaccine match [25].

## 4 Genetic characterisation of the serotype O viruses

### 4.1 Full capsid sequence analysis

The full capsid sequences of the selected East African viruses (n = 67) were generated in this study. The capsid sequences of the other eleven viruses were reported in a previous study [9 and Supplementary Table 1]. The capsid sequences of the remaining two isolates (O/EA/2009 and O/ETH/03/2012) used in this study could not be generated, either because of problems in amplifying the capsid encoding sequences or having ambiguities at more than 12 nt positions that could not be resolved even after repeated attempts. In addition, 20 capsid sequences available in the web were also included in the analysis making a total of 98 sequences from East Africa. All the sequences were 2202 nt long except O/KEN/100/2010 (EA-4 lineage) which had a 3-nt insertion (AAA) at position 1706–1708 of P1 leading to the insertion of an aa (E, glutamic acid) at position 46 of the VP1 which has been reported to be an antigenic site (Site 3) [27]. Interestingly, a historic virus O/UGA/17/98 belonging to the same lineage also has the same amino acid insertion at the same position.

Compared to the only established v/s of the region, O/KEN/77/78, the variation at nt level was 0.1% (O/KEN/125/2009) to 14.1% (O/TAN/44/2009), and 0.3% (O/KEN/125/2009) to 5.9% (O/UGA/18/2007) at aa level. Similarly, compared to the oldest viral sequence employed in our analysis, O/UGA/1/75, the variation at nt level was 8.2% (O/UGA/6/76) to 14.4% (O/NIG/15/2009), and 2.9% (O/UGA/6/76) to 5.4% (O/UGA/18/2007 and O/EGY/3/2008) at aa level. The sequence dataset was analysed by the Z-test for evidence of evolutionary selection using MEGA 6, but this was not found (P>/1.0) which probably explains why the serotype O vaccines are broadly cross-reacting.

**Phylogenetic analysis:** The 78 capsid sequences generated in this study/our previous study were used for phylogenetic analysis. In addition, 20 capsid sequences of East African type O viruses available on the web were also included in the analysis. The analysis revealed that most of the East African serotype O viruses belong to the EA topotype (1 −4), however some ME-SA topotype viruses of PanAsia-2 lineage are also circulating in Egypt and Libya, probably because of the trade links with the Middle East countries (Fig. 1B).

**Rate of nucleotide substitution per site:** From the BEAST analysis, using an uncorrelated exponential relaxed molecular clock, the rate of substitution of all the nt changes in the capsid coding region of the serotype O viruses from East Africa was estimated to be 2.16 × 10^−3^/site/year (95% HPD 1.15 × 10^−3^ to 2.75 × 10^−3^). This is similar to the report of Balinda and colleagues [6] for East African serotype O FMD viruses from 1964 to 2008 (2.7 × 10^3^ substitutions/site/year) and also by others in case of serotype O FMD viruses [28], [29], [30], [31]. The common ancestor of these viruses could have existed about 100.1 years ago, i.e. 1916 (95% HPD 66.6 to 139.3 years).

**Fig. 2:**
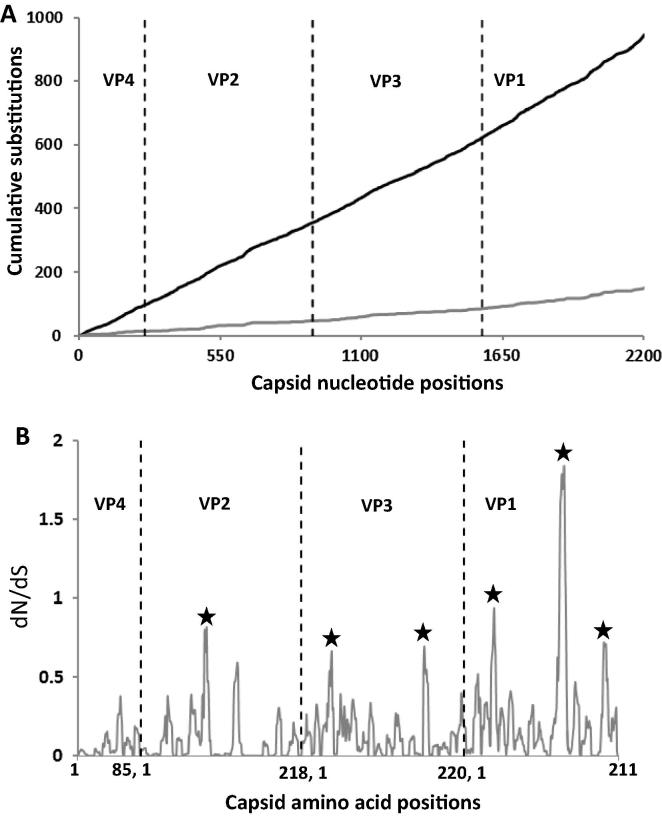
(A) Cumulative changes in capsid coding regions of serotype O East African isolates. The black line indicates total cumulative nucleotide changes, the grey line - cumulative amino acid changes. (B) dN/dS of capsid of serotype O East African isolates. The vertical black dotted line indicates gene junctions. The areas with high dN/dS ratio are marked with asterisk.

**Serotype O East African viruses:** An unbiased analysis of the capsid nucleotide sequences (VP1-4) of the 98 East African serotype O viruses revealed 1409 nt substitutions at 946 sites distributed across the region (Fig. 2A). Out of these, 87.2% of nt substitutions were found to be synonymous (silent) and 12.8% were non-synonymous (non-silent). One hundred and forty-one (1 4 1) sites were identified to have been substituted three times and 204 sites were substituted twice. At 14 sites, all the three bases of the codon were found to be mutated in the same virus encoding 3–9 different aa (Table 2A). Out of the 14 sites with three nt substitutions (encoding 3–9 aa residues), two were present in VP2, three in VP3 and nine in VP1 (Table 2A). Similarly, at nine sites, all the three bases of the codon were found to be mutated (two in VP2 and seven in VP1), but in different viruses, encoding 3–5 different aa (Table 2A).

**Table 2A:** Capsid positions where changes in all three nucleotide positions of the codon were observed. Numbers shown in parenthesis indicates number of isolates with the changes. nt: nucleotide.

Capsid protein positions	VP2	VP3	VP1
Changes in all 3 nt positions of the codon in the same virus	133 (>10), 137 (1)	134 (1), 174 (>10), 219 (6)	21 (10), 47 (1), 96 (1), 137 (4), 138 (>10), 139 (>10), 140 (10), 141 (>10), 198 (1)
Changes in all 3 nt positions of the codon, but in different viruses	39, 132	–	4, 24, 45, 95, 133, 144, 158

**Fig. 4:**
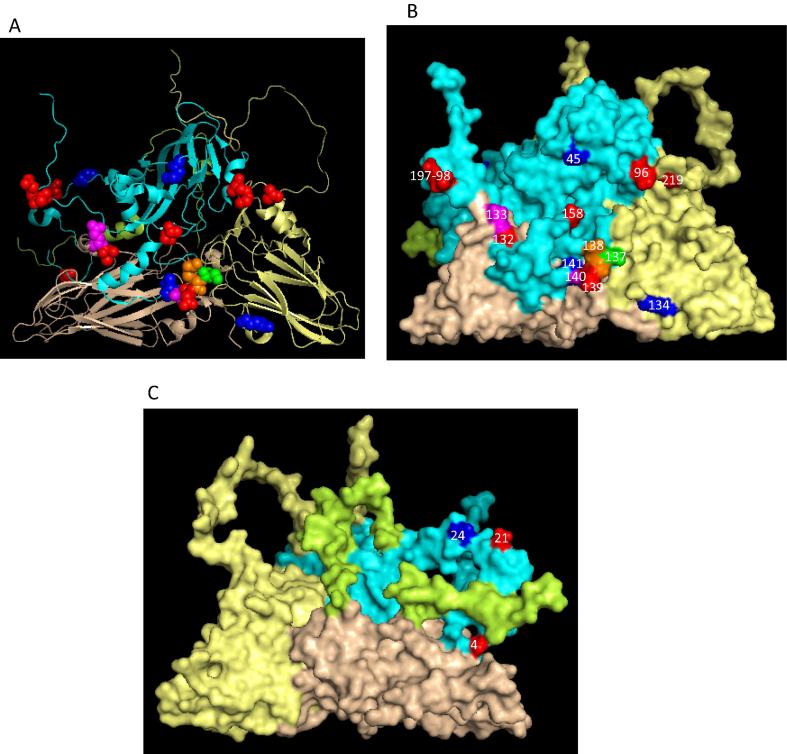
3-D structure of O BFS reduced protomer (1FOD, reduced) showing capsid position where multiple amino acid substitution occurred. The chain of VP1 is shown in cyan, VP3 – pale yellow, VP2 – wheat, VP4 – limon. The residues with four different aa substitutions are shown as red, five as blue, six as green, seven as magenta and nine as orange spheres. (A) – cartoon, (B) – external surface, (C) – internal surface. (For interpretation of the references to colour in this figure legend, the reader is referred to the web version of this article.)

The analysis of the capsid aa residues of O East African viruses revealed 285 substitutions at 149 sites across the capsid (Fig. 2A) with some sites having 2–9 alternative aa (Table 2B and Fig. 4A–C). Interestingly, sequences for VP1-138 encoded nine different aa, and VP2-133 and VP1-140 encoded seven different aa each, and exhibited nt changes at all the three positions within the codon. The non-synonymous nt substitutions were not equally distributed across the capsid coding regions: there were several local areas where the dN/dS ratio was higher than in other parts of the sequence alignment (Fig. 2B). Two regions in VP3 (42 and 168), one region in VP2 (87–90) and three regions in VP1 (40–45, 131–140 and 192–195) had dN/dS ratio of >0.65, indicative of sites under positive selection. Out of these regions the residues in VP1 133–138 had the highest dN/dS ratio (>1.5) indicating strongest selection pressure in case of East African serotype O viruses. This region has also been reported to be under strong selection pressure in case of Indian type O viruses [24] and type A viruses from the Middle East [10]. These residues are in the G-H loop, close to the integrin binding receptor motif, RGD, and forms neutralising antigenic site 1. In serotype A changes in this region has been reported to be associated with changes in the antigenicity of the virus [10], [32].

**Table 2B:** Capsid positions where multiple amino acid substitutions were observed. Numbers shown in parenthesis indicates No. of isolates with the substitution at that position.

Capsid protein positions	VP4	VP2	VP3	VP1
3 alternative aa	73 (4)	39 (7), 137 (7)	65 (6), 174 (16), 195 (6)	46 (7), 47 (4), 95 (3), 133 (11), 142 (20), 144 (3), 174 (16)
4 alternative aa	–	132 (11)	219 (16)	4 (27), 21 (24), 96 (55), 139 (56), 158 (35), 197 (37), 198 (19)
5 alternative aa	–	–	134 (13)	24 (25), 45 (50), 141 (42)
6 alternative aa	–	–	–	137 (21)
7 alternative aa	–	133 (25)	–	140 (54)
9 alternative aa	–	–	–	138 (61)

**Fig. 3:**
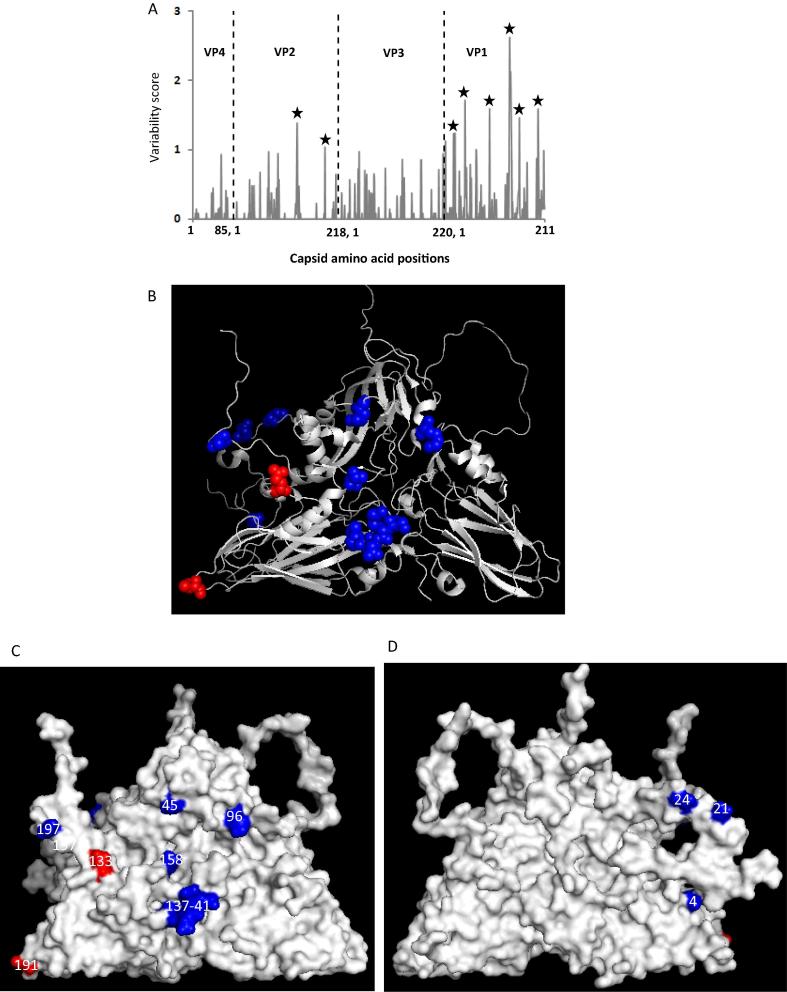
(A) Capsid amino acid variability of serotype O East African isolates. The vertical black dotted line indicates gene junctions. The areas with a variability score >1 are marked with asterisk. (B–D) 3-D structure of O BFS reduced protomer (1FOD, reduced) with highly variable capsid amino acid residues (with a score of more than 1) highlighted.VP1 residues - blue, VP3 - red, VP2 - green; (B) - cartoon, (C) – external surface, (D) – internal surface. (For interpretation of the references to colour in this figure legend, the reader is referred to the web version of this article.)

**Amino acid variability of the capsid of the East African serotype O viruses:** The deduced aa sequence of the capsid of the East African serotype O viruses were analysed further to study aa variability across the capsid. A total of 14 residues (two in VP2 and 12 in VP1) with a variability score greater than 1 were observed indicating that over 85% of the residues with high variability scores were present in VP1 (Fig. 3A). All these residues were found to be surface-exposed except VP1 4, 21 and 24 (Fig. 3B–D). Of these, VP2 133 has never been reported to be of antigenically important, however it is located close to VP2 134 that has been reported to strongly influence the binding of neutralising antigenic site 2 mAbs in serotype O FMDV [13]. Similarly VP2 191 has been recently shown to be linked to serotype O and A antigenic site 2 using a reverse genetics approach [33], [34]. VP1 45 involving antigenic site 3 and VP1 137–141, 197 involving antigenic site 1 have been reported to be critical in serotype O mar-mutant studies [27], [35]. In addition neutralising antigenic site 2 has been reported to be immunodominant in the polyclonal response of serotype O FMD-vaccinated animals followed by site 1 [36]. It therefore, appears that the changes in these residue positions could be tolerated as it has not altered the antigenicity of these viruses greatly. However continuous monitoring of the field isolates needs to be carried out to identify emergence of antigenic variants in future.

In summary, the serotype O vaccine strains used in this study are a good match with the circulating field isolates in East Africa. This indicates that serotype O vaccine strains are broadly cross-reactive and, therefore could be used as vaccines to control the disease in the region. In addition to the locally available vaccine, O/KEN/77/78, internationally available commercial vaccines like O/PanAsia-2 and O/Manisa could also cater to the needs of the region. There is always a risk of introduction of new topotypes/lineages of the viruses to the African countries as exemplified by the introduction of A-Iran-05 viruses and O-Ind-2001d viruses in Libya in 2009 and 2013, respectively, and their subsequent spread to other North African countries. In addition, most of the countries in the region are preparing to enter and/or entering to the FMD Progressive Control Pathway (PCP) as described by OIE/FAO, and would require a robust matching vaccine in the initial stage of the program. Therefore, close monitoring of the outbreak strains in the region along with regular vaccine matching studies is crucial to identify emergence of antigenic variants and also to evaluate the suitability of v/s for use in FMD control programmes in the region. The need to develop a new v/s should also be identified in a timely fashion to prevent future outbreaks.
