# Supplementary material for: Genetic and antigenic characterization of serotype O FMD viruses from East Africa for the selection of suitable vaccine strain
Source: Vaccine. 2017 Dec 14;35(49Part B):6842–9. doi: 10.1016/j.vaccine.2017.10.040 (PMC5722052; doi:10.1016/j.vaccine.2017.10.040)
Supplement: Supplementary data 1 [file mmc1.docx]

Supplementary Table 1: List of serotype O FMD viruses used in this study. SH72: Sharqia-72; PA: PanAsia; NK- not known; ND- not done.

| **Number** | **FMDV serotype O** | **Toptype (Strain)** | **Geographic origin, Country** | **Host** | **Date collected** | **Accession No.** |
| --- | --- | --- | --- | --- | --- | --- |
| 1 | O/KEN/77/1978 | EA-1 | Nakuru, Kenya | Cattle | 1978 | KP202877 |
| 2 | O/ETH/02/1993 | EA-3 | Sub. Shenge village, Gojam region, Ethiopia | NK | 16/08/1993 | KJ831667 |
| 3 | O/ETH/30/1994 | EA-3 | Seyo village, Wellega region, Ethiopia | NK | 15/08/1994 | KJ831668 |
| 4 | O/EA/2002 | EA-2 | Nakasongola district, Uganda | NK | 2002 | KJ415246 |
| 5 | O/ETH/03/2004 | EA-3 | Not specified, Ethiopia | CATTLE | 2004 | KJ831670 |
| 6 | O/TAN/02/2004 | EA-2 | Kibaha District, Pwani Region, Tanzania | CATTLE | 2004 | KJ831706 |
| 7 | O/UGA/18/2004 | EA-2 | Kasubi village, Kampala, Uganda | CATTLE | 23/03/2004 | this study |
| 8 | O/SUD/01/2005 | EA-3 | Not specified, Sudan | CATTLE | 03/01/2005 | this study |
| 9 | O/SUD/02/2005 | EA-3 | Not specified, Sudan | CATTLE | 03/01/2005 | this study |
| 10 | O/SUD/03/2005 | EA-3 | Not specified, Sudan | CATTLE | 09/01/2005 | this study |
| 11 | O/EGY/10/2006 | ME-SA (SH72) | Not specified, Egypt | CATTLE | 01/01/2006 | this study |
| 12 | O/ETH/54/2006 | EA-3 | Not specified, Ethiopia | CATTLE | 2006 | this study |
| 13 | O/ETH/01/2007 | EA-3 | Not specified, Ethiopia | CATTLE | 08/02/2007 | this study |
| 14 | O/SOM/01/2007 | EA-3 | Not specified, Somalia | CATTLE | 22/12/2007 | this study |
| 15 | O/SOM/02/2007 | EA-3 | Not specified, Somalia | CATTLE | 23/12/2007 | this study |
| 16 | O/SOM/04/2007 | EA-3 | Not specified, Somalia | CATTLE | 20/12/2007 | this study |
| 17 | O/UGA/18/2007 | EA-2 | Not specified, Uganda | CATTLE | 03/07/2007 | this study |
| 18 | O/EGY/03/2008 | ME-SA (SH72) | Not specified, Egypt | CATTLE | 03/09/2008 | this study |
| 19 | O/EGY/07/2008 | ME-SA (SH72) | Not specified, Egypt | CATTLE | 25/12/2008 | this study |
| 20 | O/ETH/29/2008 | EA-3 | Not specified, Ethiopia | CATTLE | 07/09/2008 | this study |
| 21 | O/ETH/31/2008 | EA-3 | Not specified, Ethiopia | CATTLE | 17/12/2008 | this study |
| 22 | O/KEN/37/2008 | EA-1 | Not specified, Kenya | CATTLE | 01/12/2008 | this study |
| 23 | O/SUD/04/2008 | EA-3 | Not specified, Sudan | CATTLE | 11/02/2008 | KJ831704 |
| 24 | O/SUD/08/2008 | EA-3 | Not specified, Sudan | CATTLE | 09/05/2008 | KJ831705 |
| 25 | O/TAN/16/2008 | EA-2 | Morogoro, Tanzania | CATTLE | 25/09/2008 | this study |
| 26 | O/EGY/06/2009 | ME-SA (SH72) | Not specified, Egypt | CATTLE | 22/01/2009 | this study |
| 27 | O/EGY/17/2009 | ME-SA (SH72) | Not specified, Egypt | CATTLE | 13/04/2009 | this study |
| 28 | O/EGY/23/2009 | ME-SA (SH72) | Not specified, Egypt | CATTLE | 19/04/2009 | this study |
| 29 | O/EGY/29/2009 | ME-SA (SH72) | Not specified, Egypt | SHEEP | 04/05/2009 | this study |
| 30 | O/ETH/03/2009 | EA-3 | Not specified, Ethiopia | CATTLE | 22/01/2009 | this study |
| 31 | O/ETH/11/2009 | EA-3 | Not specified, Ethiopia | CATTLE | 23/01/2009 | this study |
| 32 | O/ETH/39/2009 | EA-3 | Not specified, Ethiopia | NK | 26/03/2009 | this study |
| 33 | O/ETH/44/2009 | EA-3 | Not specified, Ethiopia | NK | 19/06/2009 | this study |
| 34 | O/ETH/49/2009 | EA-3 | Not specified, Ethiopia | CATTLE | 01/01/2009 | this study |
| 35 | O/EA/2009 | EA-2 | Not specified, Kenya | CATTLE | 01/02/2009 | ND |
| 36 | O/KEN/125/2009 | EA-1 | Nairobi, Kenya | CATTLE | 17/09/2009 | this study |
| 37 | O/NIG/15/2009 | EA-3 | Federal Lowest Area Joss, Plateau State, Nigeria | Cattle | 25/08/2009 | this study |
| 38 | O/SUD/01/2009 | EA-3 | Redwan, Omdurman, Khartoum, Sudan | CATTLE | 13/12/2009 | this study |
| 39 | O/TAN/44/2009 | EA-2 | Makete, Tanzania | CATTLE | 27/10/2009 | this study |
| 40 | O/COD/03/2010 | EA-2 | Sud-Kivu, Plaine de La Ruzizi, Dem.Rep.Congo | CATTLE | 06/10/2010 | this study |
| 41 | O/COD/04/2010 | EA-2 | Sud-Kivu, Plaine De La Ruzizi, Dem.Rep.Congo | CATTLE | 06/10/2010 | this study |
| 42 | O/ETH/07/2010 | EA-3 | Shifaro, Goba, Bale, Oromia, Ethiopia | CATTLE | 10/02/2010 | this study |
| 43 | O/EA/2010 | EA-3 | Selambar, Kucha, Walista, SNNP, Ethiopia | CATTLE | 12/02/2010 | this study |
| 44 | O/KEN/100/2010 | EA-4 | Eldoret West, Kenya | CATTLE | 28/04/2010 | this study |
| 45 | O/KEN/137/2010 | EA-2 | Transmara, Kenya | CATTLE | 06/07/2010 | this study |
| 46 | O/LIB/04/2010 | ME-SA (PA) | Tajura, Tajura, Tripoli, Libya | CATTLE | 22/12/2010 | this study |
| 47 | O/SUD/01/2010 | EA-3 | Hilat Kuku, Khartoum, Sudan | CATTLE | 11/01/2010 | this study |
| 48 | O/SUD/02/2010 | EA-3 | Keriab, Khartoum North, Khartoum, Sudan | CATTLE | 12/01/2010 | this study |
| 49 | O/ZAM/01/2010 | EA-2 | Mbala, Zambia | CATTLE | 18/10/2010 | this study |
| 50 | O/ZAM/04/2010 | EA-2 | Mbala, Zambia | CATTLE | 18/10/2010 | KJ831719 |
| 51 | O/ERI/01/2011 | EA-3 | Betmekae, Maekel region, Eritrea | CATTLE | 16/11/2011 | this study |
| 52 | O/ERI/02/2011 | EA-3 | Asmara, Maekel region, Eritrea | CATTLE | 17/11/2011 | this study |
| 53 | O/ERI/04/2011 | EA-3 | Adi-Segudo, Eritrea | CATTLE | 17/11/2011 | this study |
| 54 | O/ERI/08/2011 | EA-3 | Asmara, Maekel region, Eritrea | PIG | 05/12/2011 | this study |
| 55 | O/ETH/05/2011 | EA-3 | Sodo, Sodo, SNNP, Ethiopia | CATTLE | 05/10/2011 | this study |
| 56 | O/ETH/09/2011 | EA-3 | Sodo, Sodo, SNNP, Ethiopia | CATTLE | 20/10/2011 | this study |
| 57 | O/ETH/10/2011 | EA-3 | Uraga, Gujii, Oromia, Ethiopia | CATTLE | 20/10/2011 | this study |
| 58 | O/ETH/13/2011 | EA-3 | Adama, East Shewa, Oromia, Ethiopia | CATTLE | 31/10/2011 | this study |
| 59 | O/ETH/26/2011 | EA-3 | Shire, Samite Seharti, Tigray, Ethiopia | CATTLE | 10/11/2011 | this study |
| 60 | O/ETH/29/2011 | EA-3 | Debre-Zeit, Ethiopia | PIG | 15/07/2011 | this study |
| 61 | O/ETH/38/2011 | EA-3 | Adama, Ethiopia | CATTLE | 04/10/2011 | this study |
| 62 | O/ETH/42/2011 | EA-3 | Sidama, Ethiopia | CATTLE | 08/10/2011 | this study |
| 63 | O/ETH/48/2011 | EA-3 | Debre-Zeit (EMDIT), Ethiopia | CATTLE | 14/10/2011 | this study |
| 64 | O/KEN/01/2011 | EA-2 | Thika West, Kenya | CATTLE | 12/01/2011 | this study |
| 65 | O/LIB/25/2011 | ME-SA (PA) | Bir Muammar, Zawiyah, West Province, Libya | CATTLE | 21/12/2011 | this study |
| 66 | O/LIB/29/2011 | ME-SA (PA) | Abuesa, Zawiyah, West Province, Libya | CATTLE | 24/12/2011 | this study |
| 67 | O/LIB/33/2011 | ME-SA (PA) | Sudaim, Zawiyah, West Province, Libya | CATTLE | 25/12/2011 | this study |
| 68 | O/SUD/09/2011 | EA-3 | Mwelain, Omdurmay, Khartoum, Sudan | CATTLE | 03/03/2011 | this study |
| 69 | O/SUD/11/2011 | EA-3 | Mwelain, Omdurmay, Khartoum, Sudan | CATTLE | 03/03/2011 | this study |
| 70 | O/ETH/03/2012 | EA-3 | E.Belesa, South Gondar, Amhara, Ethiopia | CATTLE | 03/02/2012 | ND |
| 71 | O/ETH/04/2012 | EA-3 | BLD Special Zone, Amhara, Ethiopia | CATTLE | 09/02/2012 | this study |
| 72 | O/ETH/07/2012 | EA-3 | Raya Azabo, Southern Zone, Tigray, Ethiopia | CATTLE | 17/02/2012 | this study |
| 73 | O/ETH/11/2012 | EA-3 | Awabel, East Gojam, Amhara, Ethiopia | CATTLE/SHEEP | 20/05/2012 | this study |
| 74 | O/ETH/12/2012 | EA-3 | Awabel, East Gojam, Amhara, Ethiopia | CATTLE/SHEEP | 20/05/2012 | this study |
| 75 | O/ETH/15/2012 | EA-3 | Ethiopia | NK | 20/06/2012 | this study |
| 76 | O/LIB/02/2012 | ME-SA (PA) | Kerzaz, Mesrata, West Province, Libya | NK | 01/01/2012 | this study |
| 77 | O/LIB/05/2012 | ME-SA (PA) | Al Ataya, Garaboulli, West Province, Libya | CATTLE | 12/01/2012 | this study |
| 78 | O/LIB/07/2012 | ME-SA (PA) | Sug Altholatha, Zliten, West Province, Libya | SHEEP | 17/01/2012 | this study |
| 79 | O/LIB/48/2012 | ME-SA (PA) | Al Qba, Derrnah, East Province, Libya | CATTLE | 18/02/2012 | this study |
| 80 | O/LIB/54/2012 | EA-3 | Al Hawari, Benghazi, Libya | SHEEP/GOAT | 19/04/2012 | this study |
| 81 | O/Manisa | ME-SA | Turkey | CATTLE | 1969 | AJ251477 |
| 82 | O/PanAsia-2 | ME-SA | Turkey | CATTLE | 01/01/2009 | KP202878 |
